# Morphological Measurement of the Femoral Anterior Bow in Chinese Population Based on Three-Dimensional Computed Tomography

**DOI:** 10.1155/2021/7674764

**Published:** 2021-11-05

**Authors:** Yang Liu, Aobo Zhang, Rui Cai, Hao Chen, Chen Li, Qing Han, Jincheng Wang

**Affiliations:** Department of Orthopedics, The Second Hospital of Jilin University, Changchun 130041, China

## Abstract

**Purpose:**

The femoral anterior bow is an important factor in matching a femoral implant to a femur. However, its morphology in the Chinese population has rarely been reported. In this study, a three-dimensional measurement approach was adopted to provide accurate data. The aim was to supply a reference for designing a long-stemmed femoral prosthesis that is more suitable for Chinese people.

**Methods:**

Computed tomography data were collected from both lower limbs of 96 normal volunteers and reconstructed into a three-dimensional model using Mimics software. The parameters of the femoral anterior bow were measured using medical image analysis software. Statistical analysis was conducted using independent-samples and paired-samples *t*-tests with SPSS software.

**Results:**

All the indexes showed significant sexual difference (*P* < 0.05). The minimum cross-sectional area of the femoral medullary cavity was larger in men (10.77 ± 1.53 mm) than in women (9.79 ± 1.27 mm). The distance from the position of the maximum curvature to the lower edge of the lesser trochanter was also larger in men (60.93 ± 5.81 mm) than in women (56.31 ± 2.80 mm). However, the curvature of the femoral medullary cavity centerline was larger in women (883.57 ± 249.74 mm) than in men (958.79 ± 266.61 mm). The femoral anterior bow morphological indexes of Chinese subjects were different from the published data for other populations. There were no significant differences between left and right femoral anterior bows in either sex (*P* > 0.05).

**Conclusion:**

The three-dimensional measurement approach adopted in this study is more convenient and accurate than previous conventional methods, with high repeatability. The morphological indexes of the femoral anterior bow in this research suggest that population characteristics should be taken into account. This study will provide references for the design of long-stemmed femoral prostheses in the Chinese population.

## 1. Introduction

With the aging population and the popularization of artificial hip joints, periprosthetic fractures and the femoral anterior bow have gradually gained attention. Owing to their high incidence, difficult treatment, various complications, and poor functional follow-up effects [1–4], periprosthetic fractures have become an urgent and important subject in the field of joint reconstruction. According to Moazen et al. [5, 6], revision with a long-stemmed femoral prosthesis is the most effective way to solve the problem of periprosthetic fractures. However, most long-stemmed femoral prostheses are accompanied, to various degrees, by stress shielding and stress concentration, with excessive loads on the prosthesis and surrounding bone, leading to failure of revision because of imperfect design and mismatch of the prosthesis [7]. The femoral anterior bow is one of the crucial factors that cause mismatch between the prosthesis and the femur [8].

The anatomy of the femoral anterior bow varies greatly among individuals and is related to such factors as sex, race, femur length, and rheumatoid arthritis [9, 10]. In addition, the curvature and position of the anterior bow can differ widely, and the morphology of the femoral cortex changes with age, leading the anterior bow to change at the same time [11–13]. Most of the long-stemmed femoral prostheses used in Chinese hospitals are imported, and their anatomical and design data are obtained from populations in the West. Therefore, there is an urgent need for a set of long-stemmed femoral prostheses that meet the morphological parameters of the Chinese population. As a basis for designing long-stemmed femoral prostheses, it is necessary to obtain accurate morphological data of the femoral anterior bow as a reference.

In this study, three-dimensional (3D) computed tomography (CT) reconstruction technology was used to measure the morphology of the femoral anterior bow in the Chinese population. Also, accurate morphological data of the femoral anterior bow were provided. The aim of this study was to guide the design and optimization of long-stemmed femoral prostheses to match the morphological characteristics of the Chinese.

## 2. Materials and Methods

A total of 50 male and 46 female Chinese northeast volunteers attended this study. Their lower limbs were all scanned using CT. All the tests were approved by the ethics committee of the Second Hospital of Jilin University (No. 202, 2018). This study was conducted in accordance with the principles outlined in the Declaration of Helsinki. Informed consent was obtained from all individual participants included in the study. The details of the volunteers were as follows:

1. Age: the average age of men was 29 years (range, 23–46 years) and that of women was 26 years (range, 20–39 years)
2. Height: the average height of men was 178 cm (range, 168–185 cm) and that of women was 168 cm (range, 160–178 cm)
3. Body mass index (BMI): the average BMI of men was 22.38 kg·m^−2^ (range, 19.37–27.76 kg·m^−2^) and that of women was 21.16 kg·m^−2^ (range, 16.33–26.35 kg·m^−2^)

Subjects with any of the following were excluded:

1. A history of fracture of the hip or femur
2. Congenital malformation
3. Deformation of the hip or femur

The CT scans were obtained using a TOSHIBA/Aquilion ONE scanner with the following scanning parameters: 120 kV, 80 mA, window width: 512 pixels, window height: 512, pixel size: 0.965 mm, and slice thickness: 0.5 mm.

### 2.1. Three-Dimensional Reconstruction of the Femur

**Figure 1:**
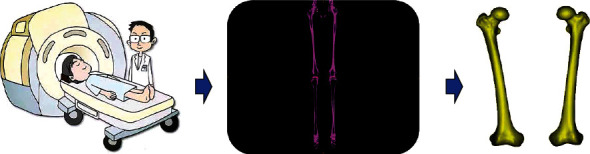
The process from CT scanning to 3D reconstruction of the femur.

In this study, the subjects' lower limbs were scanned using CT. After scanning, the CT images were exported to Mimics (v. 19.0, Materialise, Belgium) [14, 15] in digital imaging and communications in medicine (DICOM) format. Using the “threshold,” “region grow,” “spilt mask,” and “calculate 3D” functions in Mimics software, the images were reconstructed to produce three-dimensional models. The three-dimensional model was exported to Magics (v. 21.0, Materialise, Belgium) in stereolithography (STL) format (Figure 1).

### 2.2. Medullary Cavity Extraction

**Figure 2:**
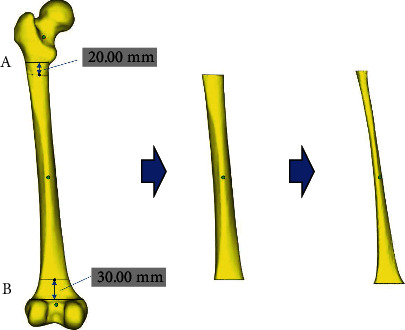
The process of medullary cavity extraction.

The STL files were imported into Magics software and placed uniformly according to anatomical position. Using the “distance” function in Magics software, point *A* (Figure 2) was determined as 20 mm away from the upper edge of the femoral medial epicondyle and lateral epicondyle, and point *B* (Figure 2) was determined as 30 mm away from the lower edge of the femoral lesser trochanter. Then, the software “cut” and “repair” functions were used to remove unnecessary parts and model the medullary cavity.

### 2.3. Fitting the Centerline of the Femoral Medullary Cavity

The medullary cavity model was subsequently exported for engineering modification in 3-matic software (v. 13.0, Materialise, Belgium). The medullary cavity was cut into approximately 300 sections along the bend, and the center points of each section were connected to form a centerline using 3-matic software. Then, the minimum section was found and its area measured. Since the cross-section of the medullary cavity is approximately circular, its diameter could be used to express its area. For this reason, the minimum cross-sectional area of the femoral medullary cavity was represented using the minimum diameter (MD).

**Figure 3:**
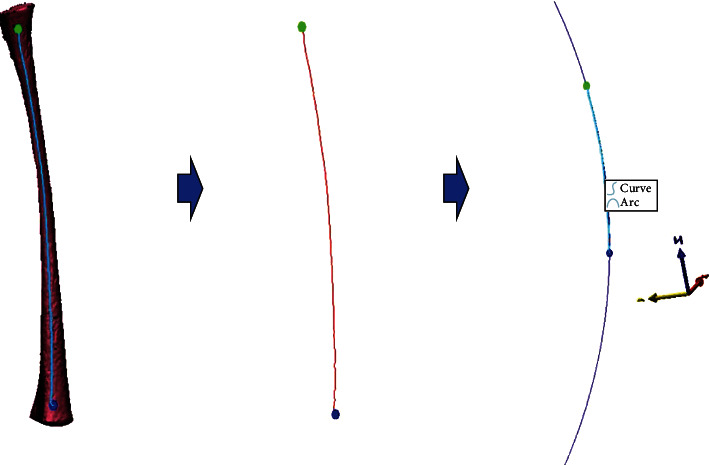
The process of fitting the centerline.

The centerline of the medullary cavity was extracted and exported in text (txt) format. The parameters were modified so that the file could be imported into UG NX software (v. 12.0, Siemens, Germany). The fitting curve function of UG NX was used to fit the scattered points to the overall curve and derive the fitted centerline for measurement. After that, the centerline could be fitted to a circle. According to Bruns et al. [16], the femoral anterior bow could be considered part of a virtual circle, the radius of which could be considered the radius of the femoral curvature (RFC). Thus, the radius of the previous fitted circle from the centerline could be considered the RFC. Then, the maximum curvature position on the centerline (the vertex of the anterior femoral bow), defined as point *P*, was determined, and the distance from point *A* to point *P* (Dist.) and the central angle of $\overset{⌢}{\text{AP}}$ in the virtual circle (angle *α*) were measured (Figure 3).

### 2.4. Measurement of the Femoral Anterior Bow Morphological Indexes

**Figure 4:**
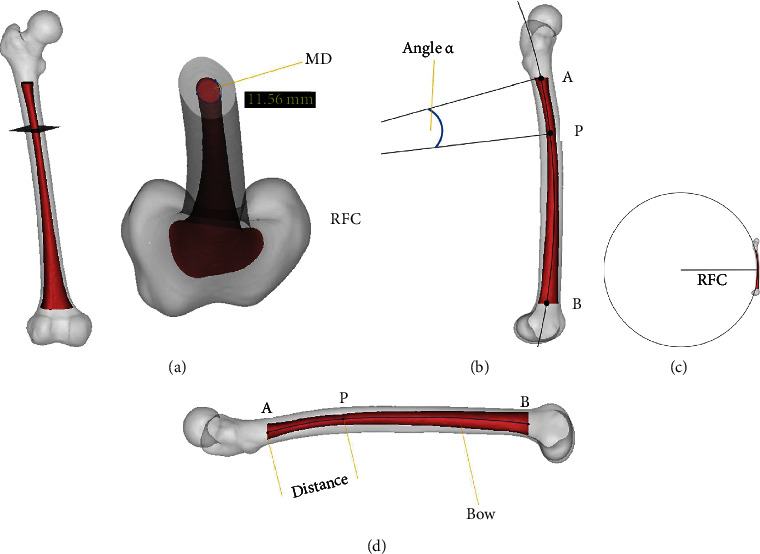
Indexes measured in this study: (a) the cross-section is approximately a circle, and its minimum diameter is measured as 11.56 mm (MD); (b) center angle corresponding to $\overset{⌢}{\text{AP}}$ in the virtual circle (angle *α*); (c) radius of the femoral curvature (RFC); (d) distance between point *A* and point *P* (Dist.).

In total, four indexes were measured (Figure 4): the MD, the RFC, the distance from point *A* to point *P* (Dist.), and the central angle corresponding to $\overset{⌢}{\text{AP}}$ in the virtual circle (angle *α*).

### 2.5. Statistical Analysis

SPSS software (v. 21.0, IBM, USA) was used to conduct statistical analysis. The normal distribution of each index was tested. Each measurement was made by two researchers, and each index was measured twice by each researcher. One researcher is an attending physician, and the other is a resident physician. These two researchers have been trained in conducting anatomic and radiologic research. The average of the four measurements was taken as the final result, and the data were presented as the mean ± standard deviation of each index. Interobserver and intraobserver reliability was estimated using the intraclass correlation coefficient (ICC). The comparisons between the left and right femoral anterior bows in either men or women in this study were analyzed using a paired-samples *t*-test. The comparison between male and female subjects of this study was analyzed using an independent-samples *t*-test. Statistical significance was considered for *P* < 0.05.

## 3. Results

### 3.1. Interobserver and Intraobserver Reliability

The ICC of each variable was in the range of its 95% confidence interval (Table 1). None of the tests was statistically significant for any of the variables; therefore, there were no differences between or within observers. Statistically acceptable coefficients of reproducibility could be obtained.

### 3.2. Measurement of the Minimum Cross-Sectional Area of the Femoral Medullary Cavity

In this study, the minimum cross-section of the femoral medullary cavity was regarded as an approximate circle, and the MD was used as the measurement index. The MD was normally distributed in the entire cohort, as well as in subgroups of men and women. The MD was significantly larger in men (10.77 ± 1.53 mm) than in women (9.79 ± 1.27 mm) (*P* < 0.05) (Table 2). No significant difference between left and right femoral anterior bows in either men or women was identified (*P* > 0.05) (Table 3).

### 3.3. Measurement of the Centerline Curvature of the Femoral Medullary Cavity

In this study, the RFC was used to represent the centerline curvature of the femoral medullary cavity. The RFC was normally distributed in the entire cohort as well as in subgroups of men and women. The RFC was significantly larger in men (958.79 ± 266.61 mm) than in women (883.57 ± 249.74 mm) (*P* < 0.05) (Table 2). In other words, the centerline curvature in men was smaller than that in women. No significant difference between left and right femoral anterior bows in either men or women was identified (*P* > 0.05) (Table 3).

### 3.4. Measurement of the Position of the Maximum Curvature of the Femoral Anterior Bow

Both Dist. and angle *α* were used as the measurement indexes of the position of the maximum curvature; Dist. and angle *α* were normally distributed in the entire cohort as well as in subgroups of men and women. Both Dist. and angle *α* were significantly larger in men than in women (*P* < 0.05) (Table 2). No significant difference between left and right femoral anterior bow in either men or women was identified (*P* > 0.05) (Table 3).

## 4. Discussion

To the best of our knowledge, only a small number of studies have been conducted on the femoral anterior bow, compared with research on the medullary cavity of the proximal femur. Measurement methods of the femoral anterior bow from previous studies are mainly divided into three levels: measurements from physical specimens, X-radiography, and CT.

A representative method of measuring physical specimens was proposed by Egol et al. [10]. In this study, the existing bone specimens in the American Museum of Natural History in New York and the Cleveland Museum of Natural History in Ohio were uniformly fixed and photographed. Using the two-dimensional photographs, Egol et al. made an arc based on the lower trochanter, the upper femoral condyle, and the midpoint of the two positions. The curvature of the femoral anterior bow was represented by the curvature of the arc. This measurement approach needed stricter requirements for the fixed system, and the system error was relatively large. However, it provided a simple and direct methodological reference and paved the way for subsequent X-ray and CT studies.

The clinical X-ray measurement method of the femoral anterior bow is as follows. First, the long axis of the femoral condyle is found on the femoral lateral radiograph. Second, a vertical line through the midpoint of the line is drawn. Third, the axis of the upper third of the femur is marked. Finally, the angle between the two lines is measured. The femoral anterior bow angle of normal adults is given as 10.7° ± 5.6° [17]. The radius of the femoral curvature reported by Bong et al. [18] using the X-ray measurement method was 120 ± 36 cm; Tang et al. [12] divided the full length of the femur into three parts on the X-ray film and measured the radius of curvature of each part. A possible shortcoming of this research is that these analyses are of two-dimensional images; the actual curvature occurs in three dimensions. Moreover, X-ray films that are easily obtained from the clinic will exhibit variability in structural parameters, owing to differences in posture and tube projection angles. These factors may be the main reason for the deviation of the X-ray measurement results from the actual results.

The CT reconstruction method used to measure the femoral anterior bow has the advantages of accuracy, convenience, and high repeatability [19, 20]. Maratt et al. [9] reported that they placed the three-dimensional image reconstructed by CT according to the aforementioned physical placement method. Next, they measured the corresponding distance on the two-dimensional image to determine the parameters related to the femoral anterior bow. It is worth noting that a multipoint connection between the inner cortex and the centerline of the medullary cavity was drawn on the two-dimensional photograph of the reconstructed femur for curvature fitting in that study [9]. Lu et al. [13] measured the femoral curvature in three equal parts on the femoral reconstruction model. The measurement of the femoral cavity through CT has gained some clinical applications. Zhang et al. [21] used CT to assess the morphology of the femoral medullary canal in subjects with developmental dysplasia of the hip with the intent of improving the design of femoral stems in total hip arthroplasty. Yao et al. [22] used 3D designing osteotomy and implantation of the femoral component to treat proximal femur fibrous dysplasia patients combined with hip joint osteoarthritis. In 2014, Buford et al. [23] proposed, for what we understand to be the first time, a method of determining the medullary cavity for measurement. They used the filling replacement method to identify the medullary cavity. However, their study still used three-point fitting measurements of the outer cortex and the surface of the medullary cavity. The aforementioned measurement methods are not true three-dimensional measurements, because the most accurate and effective curvature of the femoral anterior bow should be the curvature of the line connecting the midpoints of each section of the medullary cavity, rather than the multipoint connection on the surface of the medullary cavity or the inner and outer sides of the cortex. In our study, the extracted medullary cavity segment was cut into about 300 sections along the bend, and the center point of each section was connected to form a centerline in 3-matic software. Only in this way can we achieve a truly three-dimensional measurement. Since we have used a theoretically more accurate method to fit the femoral anterior bow, the morphological index that we obtained will be closer to reality than other studies used in the past.

In our study, there was no significant difference between the left and right femoral anterior bow in either men or women (*P* > 0.05), which was consistent with the conclusions of previous research [10, 17, 24]. Therefore, when customizing the long-stemmed femoral prosthesis for particular patients, the morphological data of the contralateral femoral anterior bow could be used as an important reference. The four morphological indexes of the femoral anterior bow (MD, RFC, Dist., and angle *α*) measured in this study were all significantly larger in men than women (*P* < 0.05). This indicates that the minimum cross-sectional area of the femoral medullary cavity of men is larger than that of women, while the curvature of the femoral anterior bow is smaller than that of women. Moreover, the position of the maximum curvature of the femoral anterior bow was relatively lower than that in women. Thus, we should pay attention to the sexual difference of the femoral anterior bow in selecting or designing long-stemmed femoral prostheses.

Other researchers have made similar morphological measurements of the femoral anterior bow before. Egol et al. [10] measured the curvature radius of the femoral anterior bow of Black and White Americans using bone specimens. Their results showed that the curvature radii of Black men, Black women, White men, and White women were 132 cm, 133 cm, 119 cm, and 105 cm, respectively; the X-ray measurement of the average curvature radius of the femoral anterior bow was 120 cm ± 36 cm. Chantarapanich et al. [25] used cadaver specimens to measure the curvature radius of the femoral anterior bow in a Thai population using three-dimensional reconstruction and obtained an average RFC of 895.46 ± 238.06 mm. However, the average RFCs for men, women, and all subjects in this study were 958.79 ± 266.61 mm, 883.57 ± 249.74 mm, and 922.74 ± 260.05 mm, which are significantly smaller than the curvature radius of the femoral anterior bow of the Americans but larger than that of the Thais, who were also Asians. These results show that there are great differences in the morphological measurement indexes of the femoral anterior bow in different ethnic groups and in people in different regions. Therefore, the long-stemmed femoral prosthesis designed for the Western population is not suitable for the Chinese population.

The impact of the femoral anterior bow on implants is mainly reported for intramedullary nails, while reports on long-stemmed revision prostheses are few, and most of them are clinical case analyses. Through a retrospective review, Bazylewicz et al. [26] concluded that intramedullary nails with a curvature radius of 180 cm had a smaller probability of piercing the femoral anterior cortex. Collinge and Beltran [27] used X-ray films to compare the centerline of intramedullary nails with curvature radii of 150 cm and 200 cm at the midline of the medullary cavity; their results confirmed the importance of the implant curvature radius for the success of implantation. Egol et al. [10] measured the curvature radius of eight types of intramedullary nails and three types of long-stemmed prostheses. The measured RFC ranges of intramedullary nails and femoral long-stemmed prostheses were 186–300 cm and 127–200 cm, which were significantly larger than the average Chinese RFC measured in this study, 922.74 ± 260.05 mm. At present, most of the femoral long-stemmed prostheses used in China are imported. The prostheses produced by leading domestic medical device companies are also designed to imitate Western prostheses, and there is a lack of localized research. Therefore, designing a femoral long-stemmed prosthesis for Chinese needs to be put on the agenda.

This study has several limitations. The subjects in this study are all from northeast China; this situation does not allow us to determine whether there are regional differences in femoral anterior bow indexes within China. The sample size of 96 seems inadequate, compared with most conventional studies that use cadaver specimens. If the same method can be used to conduct large-scale femoral data statistics in different regions in China, it will be of great significance to the design and application of domestic femoral long-stemmed prostheses.

## 5. Conclusions

This study used CT three-dimensional reconstruction technology to make accurate morphological measurements of the femoral anterior bow of the Chinese population, provides morphological data of the normal femoral anterior bow of the Chinese, and reveals that the morphological data of the femoral anterior bows of different races and different sexes have significant differences. It supplies an important basis for guiding the design and optimization of femoral long-stemmed prostheses that conform to the morphological characteristics of the Chinese.

## Figures and Tables

**Table 1:** Interobserver and intraobserver reliability estimated by intraclass correlation coefficient (ICC).

Index	ICC A1−A2	ICC B1−B2	ICC A1−B1	ICC A2−B2
MD (mm)	0.966	0.957	0.932	0.929
RFC (mm)	0.948	0.955	0.943	0.951
Dist. (mm)	0.969	0.973	0.931	0.938
Angle *α* (°)	0.985	0.957	0.917	0.926

MD: minimum diameter; RFC: radius of the femoral curvature; Dist.: distance from point *A* to point *P*; angle *α*: central angle corresponding to $\overset{⌢}{\text{AP}}$.

**Table 2:** Descriptive statistics of the femoral anterior bow.

Index	Men (*n* = 100)		Women (*n* = 92)		*P*	Total (*n* = 192)	
	Mean ± SD	Range	Mean ± SD	Range		Mean ± SD	Range
MD (mm)	10.77 ± 1.53	7.16–14.60	9.79 ± 1.27	6.56–12.42	≤0.001	10.30 ± 1.48	6.56–14.60
RFC (mm)	958.79 ± 266.61	509.91–1822.58	883.57 ± 249.74	452.74–1584.65	0.046	922.74 ± 260.05	452.74–1822.58
Dist. (mm)	60.93 ± 5.81	42.62–74.67	56.31 ± 2.80	50.33–64.88	≤0.001	58.72 ± 5.14	42.62–74.67
Angle *α* (°)	5.01 ± 1.22	2.24–7.46	4.56 ± 1.35	2.15–7.49	0.016	4.79 ± 1.30	2.15–7.49

MD: minimum diameter; RFC: radius of the femoral curvature; Dist.: distance from point *A* to point *P*; angle *α*: central angle corresponding to $\overset{⌢}{\text{AP}}$.

**Table 3:** Paired-samples *t*-test of the anatomical measurements of the femoral anterior bow.

Index	Men (*n* = 50)		*P*	Women (*n* = 46)		*P*
	Right	Left		Right	Left	
MD (mm)	10.74 ± 1.50	10.75 ± 1.57	0.894	9.75 ± 1.32	9.83 ± 1.24	0.175
RFC (mm)	948.86 ± 258.22	968.72 ± 277.02	0.416	876.63 ± 246.71	890.51 ± 255.27	0.748
Dist. (mm)	60.98 ± 5.70	61.08 ± 5.74	0.899	55.89 ± 2.73	56.73 ± 2.83	0.072
Angle *α* (°)	5.09 ± 1.20	4.93 ± 1.25	0.839	4.54 ± 1.30	4.58 ± 1.41	0.380

MD: minimum diameter; RFC: radius of the femoral curvature; Dist.: distance from point *A* to point *P*; angle *α*: central angle corresponding to $\overset{⌢}{\text{AP}}$.

## Data Availability

The data supporting the findings of this study are available from the corresponding authors upon reasonable request.
